# Release of an HtrA-Like Protease from the Cell Surface of Thermophilic *Brevibacillus* sp. WF146 via Substrate-Induced Autoprocessing of the N-terminal Membrane Anchor

**DOI:** 10.3389/fmicb.2017.00481

**Published:** 2017-03-21

**Authors:** Fengtao Zhu, Xing Yang, Yan Wu, Yasi Wang, Xiao-Feng Tang, Bing Tang

**Affiliations:** ^1^State Key Laboratory of Virology, College of Life Sciences, Wuhan UniversityWuhan, China; ^2^Hubei Provincial Cooperative Innovation Center of Industrial FermentationWuhan, China

**Keywords:** autoprocessing, thermophile, heat resistance, HtrA protease, N-terminal domain, PDZ domain, calcium-binding protein

## Abstract

High-temperature requirement A (HtrA)-like proteases participate in protein quality control in prokaryotes and eukaryotes by degrading damaged proteins; however, little is known about HtrAs produced by thermophiles. HtrAw is an HtrA-like protease of thermophilic *Brevibacillus* sp. WF146. The intact form of HtrAw (iHtrAw) consisting of a transmembrane segment-containing N-terminal domain, a trypsin-like protease domain, and a C-terminal PDZ domain was produced in *Escherichia coli*. Purified iHtrAw itself is unable to cleave the N-terminal domain, but requires protein substrates to autoprocess the N-terminal domain intermolecularly, yielding a short form (sHtrAw). Mutation at the substrate-binding site in the PDZ domain affects the conversion of iHtrAw to sHtrAw. Deletion analysis revealed that the N-terminal domain is not necessary for enzyme folding, activity, and thermostability. Compared with other known HtrAs, HtrAw contains an additional Ca^2+^-binding Dx[DN]xDG motif important for enzyme stability and/or activity. When produced in an *htrA*/*htrB* double deletion mutant of *Bacillus subtilis*, iHtrAw localized predominantly to the cell pellet, and the amount of sHtrAw in the culture supernatant increased at elevated temperatures. Moreover, HtrAw increased the heat resistance of the *B. subtilis* mutant. In strain WF146, HtrAw exists in both a cell-associated intact form and a cell-free short form; an increase in growth temperature enhanced HtrAw production and the amount of cell-free short form. Release of the short form of HtrAw from the membrane may have the advantage of allowing the enzyme to freely access and degrade damaged proteins surrounding the bacterium living at high temperatures.

## Introduction

The serine protease high-temperature requirement A (HtrA) plays a pivotal role in protein quality control in prokaryotes and eukaryotes. *Escherichia coli* possesses three HtrAs (DegS, DegP, and DegQ), which represent the best-characterized HtrAs involved in removing misfolded or damaged proteins in an ATP-independent manner in the periplasm (Merdanovic et al., 2011). DegS is composed of an N-terminal domain with a transmembrane segment (TMS), a trypsin-like protease domain, and a C-terminal PDZ domain, whereas both DegP and DegQ are synthesized as a precursor containing an N-terminal signal peptide, a trypsin-like protease domain, and two C-terminal PDZ domains (Clausen et al., 2002; Hansen and Hilgenfeld, 2013). Under harsh conditions such as elevated temperatures, the inner membrane-anchored DegS is activated via binding of misfolded/unfolded outer membrane proteins (OMPs) in the periplasm, thereby specifically hydrolyzing the anti-σ factor RseA to induce σ^E^-dependent transcription of stress genes including *degP* (Danese and Silhavy, 1997; Clausen et al., 2002; Walsh et al., 2003). The signal peptide of DegP is cleaved after translocation of the enzyme across the inner membrane, and mature DegP is activated to release non-specific protease activity upon binding to misfolded/unfolded proteins under stress conditions. DegP also acts as a chaperone under non-stress conditions to protect folded OMPs from proteolysis during their transport through the periplasm and to assist in folding of periplasmic proteins such as α-amylase MalS (Spiess et al., 1999; Jiang et al., 2008; Krojer et al., 2008b). In *E. coli*, the *degQ* gene is located immediately upstream of the *degS* gene; DegQ has a high sequence identity (∼60%) to DegP and can functionally substitute for DegP under certain conditions, although the *degQ* gene is not heat-inducible (Waller and Sauer, 1996). While DegS (lacking its N-terminal TMS) forms stable trimers, DegP and DegQ are able to adopt multiple oligomeric states (e.g., trimer, hexamer, 12-mer, and 24-mer), which determine the active state of the enzyme (Wilken et al., 2004; Krojer et al., 2008b; Jiang et al., 2008; Bai et al., 2011; Sawa et al., 2011).

HtrAs of Gram-positive bacteria share the same modular domain architecture as the *E. coli* DegS but are functionally similar to the *E. coli* DegP, acting as both a protease and a chaperone to degrade or refold misfolded proteins within the cell envelope under stress conditions (Poquet et al., 2000; Jones et al., 2001; Antelmann et al., 2003; Ahn et al., 2005; Zweers et al., 2009; Chitlaru et al., 2011; Noone et al., 2012). In *Bacillus subtilis*, three genes encoding HtrAs have been identified: *htrA* (or *ykdA*), *htrB* (or *yvtA*), and *yyxA* (or *yycK*) (Noone et al., 2001). The expression of *htrA* and *htrB* is controlled by the CssRS two-component system that responds to heat and secretion stresses, whereas *yyxA* is neither heat-shock nor secretion-stress inducible (Noone et al., 2001; Darmon et al., 2002). HtrA and HtrB of *B. subtilis* facilitate the extracytoplasmic quality control and folding of secreted proteins, lipoproteins, and membrane proteins, and are thus crucial for maintaining the integrity of the bacterial cell even under non-stress conditions (Krishnappa et al., 2013, 2014). The requirement of HtrAs for the biogenesis of secreted proteins has been reported for other Gram-positive bacteria such as *Streptococcus pyogenes* (Rosch and Caparon, 2005) and *Lactococcus lactis* (Poquet et al., 2000). HtrAs also contribute to the virulence of many Gram-positive pathogens (Jones et al., 2001; Ibrahim et al., 2004; Lyon and Caparon, 2004; Ahn et al., 2005; Wilson et al., 2006; Chitlaru et al., 2011), primarily through their roles in stress resistance and survival of bacteria, as well as processing of extracellular virulence factors. Although HtrAs of Gram-positive bacteria can anchor to the outer surface of the cytoplasmic membrane through their N-terminal TMSs, some of them can be released into the culture medium in a TMS-truncated form, such as *B. subtilis* HtrA and HtrB (Antelmann et al., 2003; Zweers et al., 2009), *B. anthracis* HtrA (Chitlaru et al., 2011), and *Mycobacterium tuberculosis* HtrA2 and HtrA3 (Skeiky et al., 1999; Mohamedmohaideen et al., 2008; White et al., 2010). However, the mechanism of TMS truncation and the release of these enzymes into the culture medium remains unclear.

While HtrAs of mesophiles have been extensively studied for their roles in protein quality control under stress conditions including heat stress, little is known about HtrAs from thermophiles that naturally survive at high temperatures. Only DegQ from *Thermotoga maritima*, a hyperthermophilic Gram-negative bacterium, has been characterized in terms of its crystal structure and activation mechanism (Kim et al., 2003, 2008). To the best of our knowledge, there is no literature regarding HtrA-like protease from thermophilic Gram-positive bacteria. *Brevibacillus* sp. WF146 (previously named *Bacillus* sp. WF146) is a thermophilic bacterium with an optimal growth temperature of approximately 58°C (Wu et al., 2004). The major extracellular protease (WF146 protease) of the strain WF146 has been characterized in detail (Bian et al., 2006; Yang et al., 2008; Zhong et al., 2009; Liang et al., 2010; Zhu et al., 2013; Xu et al., 2015). We recently obtained a draft genome sequence of the strain WF146 and identified a gene encoding an HtrA (named HtrAw; GenBank accession number WP_029100535). The purpose of this study was to investigate whether HtrAw is functionally similar to its mesophilic counterpart and to probe possible adaptation mechanism of this enzyme to high temperatures. The gene of HtrAw was cloned and expressed in *E. coli* and *B. subtilis* to investigate the enzymatic properties and processing mechanism of this enzyme. The production and localization of HtrAw in the strain WF146 were examined, and the mechanism of release of membrane-anchored HtrAw into the culture medium by substrate-induced autoprocessing of the N-terminal TMS was discussed.

## Materials and Methods

### Bacterial Strains and Growth Conditions

Unless otherwise indicated, *Brevibacillus* sp. WF146 (previously named *Bacillus* sp. WF146; CCTCC AB209297) was grown in 50 ml Luria-Bertani (LB) medium in a 250-ml flask at 55°C with shaking (180 rpm). *E. coli* DH5α and JM110 strains were used for cloning, and *E. coli* BL21 (DE3) was used for protein expression. *B. subtilis* DB104 (*his nprR2 nprE18 ΔaprA3*) (Kawamura and Doi, 1984) was used as the parental strain for deleting *htrA* and *htrB* genes. *E. coli* and *B. subtilis* strains were grown at 37°C with shaking (180 rpm) in a 250-ml flask containing 50 ml LB medium supplemented with chloramphenicol (34 μg/ml), kanamycin (30 μg/ml), or ampicillin (100 μg/ml) as needed. Bacterial growth was monitored by measuring the culture optical density at 600 nm (OD_600_). *B. subtilis* strain cultures were plated on LB agar plates and incubated at 37°C overnight to determine the number of viable cells.

### Strain Construction

The *E. coli*/*B. subtilis* shuttle vector pNNB194 (Connelly et al., 2004) was used as the backbone for gene deletion constructs. Briefly, an approximately 500-bp DNA sequence upstream of the 5′ end or downstream of the 3′ end of the target gene was amplified from the *B. subtilis* DB104 genome. The two DNA fragments obtained were joined together by overlap extension PCR (Bian et al., 2006), and then the resulting DNA fragment was inserted into the *Hin*d III-*Eco*R I site of pNNB194 to generate the deletion plasmid containing approximately 1000 bp of DNA homologous to the region flanking the deletion. The primers used for gene deletion constructs are listed in Supplementary Tables S1, S2. Starting from *B. subtilis* DB104, *htrA* and *htrB* genes were deleted in a sequential manner according to the method of Connelly et al. (2004), yielding an *htrA/htrB* double deletion mutant strain, ZT1. Chromosomal deletions were confirmed by PCR analysis and DNA sequencing.

### Plasmid Construction and Mutagenesis

The genomic DNA of *Brevibacillus* sp. WF146 was prepared as previously described (Wu et al., 2004) and used as the template for PCR. The oligonucleotide sequences of primers used for plasmid construction are listed in Supplementary Table S1. The plasmid pET26b (Novagen) was used as the vector for expressing recombinant proteins in *E. coli* BL21 (DE3). The full-length gene of HtrAw was amplified from the genomic DNA by PCR using the primer pair Aw-F/Aw-R or Awb-F/Awb-R (Supplementary Table S2), and the PCR product was inserted into the *Nde* I-*Hin*d III site of pET26b to construct the expression plasmid pET26b-*iHtrAw* or pET26b-*iHtrAwb* for intact HtrAw with a C-terminal His-tag (iHtrAw) or an N-terminal His-tag (iHtrAwb). The genes encoding the N-terminal domain-deletion variant (ΔN74), the PDZ-deletion variant (ΔPDZ), the variant lacking both the N-terminal domain and the PDZ (ΔNP), and the PDZ domain (PDZ) were amplified from pET26b-*iHtrAw* by PCR using the primer pairs listed in Supplementary Table S2. The PCR products were inserted into the *Nde* I-*Hin*d III site of pET26b to generate expression plasmids for target proteins with a C-terminal His-tag. The QuikChange site-directed mutagenesis method (An et al., 2010) was employed to construct the active-site variant of iHtrAw (S249A), the substrate-binding site variant (Mut4S), and the Dx[DN]xDG motif variant (Mut3D) of iHtrAwb using the primers listed in Supplementary Tables S1, S2.

In order to produce recombinant proteins in *B. subtilis* strain ZT1 and to facilitate the DNA manipulation, a *Not* I restriction site was introduced into plasmid pHT43 (Biovector Science Lab, Inc., Beijing, China) through the QuikChange site-directed mutagenesis method (An et al., 2010) using the primer pair NotI-F/NotI-R (Supplementary Tables S1, S2), yielding the plasmid pHT43a. The gene encoding intact HtrAw was amplified from the genomic DNA by PCR using the primer pair Aw-BF/ Aw-BR, and the PCR product was inserted into the *Not* I-*Xba* I site of pHT43a to construct the expression plasmid pHT43a-*iHtrAw* (Supplementary Tables S1, S2). The sequences of all recombinant plasmids were confirmed by DNA sequencing.

### Expression, Purification, and Refolding

*Escherichia coli* BL21 (DE3) cells harboring recombinant plasmids were grown at 37°C until the OD_600_ reached approximately 0.6. Production of the recombinant protein was induced by addition of 0.4 mM isopropyl-β-D-thiogalactopyranoside (IPTG), and cultivation was continued at 30°C for approximately 6 h. The cells were harvested, suspended in buffer A (50 mM Tris-HCl, 10 mM CaCl_2_, pH 7.5), and disrupted by sonication on ice. The insoluble and soluble cellular fractions were separated by centrifuging the cell lysate at 13,000 × *g* for 10 min. The periplasmic fraction was prepared using the osmotic shock method, as suggested in the pET expression system manual (Novagen). Briefly, the cells were suspended in 30 mM Tris-HCl (pH 8.0) and 20% sucrose, and the cell suspension was supplemented with 1 mM EDTA, and then stirred slowly with a magnetic stirring bar at room temperature for 10 min. Subsequently, cells were collected by centrifugation at 10,000 × *g* for 10 min at 4°C, and then resuspended in ice-cold 5 mM MgSO_4_. The cell suspension was stirred slowly for 10 min on ice and then centrifuged at 10,000 × *g* for 10 min at 4°C. The resulting supernatant was collected and used as the periplasmic fraction. The pelleted cells were suspended in buffer A, disrupted by sonication on ice, and centrifuged at 13,000 × *g* for 10 min. The resulting supernatant was collected and used as the soluble cytoplasmic fraction.

The recombinant proteins in the soluble cellular fractions of *E. coli* cells were purified using affinity chromatography on a Ni^2+^-charged Chelating Sepharose Fast Flow resin (GE Healthcare, Little Chalfont, UK) column equilibrated with buffer A containing 20 mM imidazole. After washing the column with buffer A containing 40 mM imidazole, the His-tagged proteins were eluted with buffer A containing 300 mM imidazole and then dialyzed against buffer A overnight at 4°C. The recombinant proteins in the insoluble cellular fractions were solubilized in buffer A containing 8 M urea. After centrifugation at 13,000 × *g* for 10 min, the supernatants were subjected to affinity chromatography as mentioned above, with the addition of 8 M urea in the washing and elution buffers. The elution fractions containing denatured recombinant proteins were dialyzed against buffer A overnight at 4°C to allow for protein refolding. Protein concentrations were determined using the Bradford method (Bradford, 1976), with bovine serum albumin (BSA) as a standard. The enzyme solution was concentrated with a Micron TM-3 centrifugal filter (Millipore, Bedford, MA, USA) as needed.

*Bacillus subtilis* strain ZT1 cells harboring expression plasmids for target proteins were grown in LB medium at 37°C until the OD_600_ reached approximately 1.0. Production of recombinant protein was induced by addition of 0.4 mM IPTG, and cultivation continued for 2 h. Subsequently, cultures were continually grown at 37°C or shifted to 50°C for increasing periods of time. The number of viable cells in the culture was determined via plate counting to determine the effects of recombinant proteins on heat resistance of the bacteria.

### Enzymatic Activity Assay

Unless otherwise indicated, the azocaseinolytic activity of the enzyme was assayed at 55°C for 1 h in 200 μl of reaction mixture containing 0.25% (wt/vol) azocasein (Sigma, St. Louis, MO, USA) and 100 μl of enzyme sample in Buffer A. The reaction was terminated by addition of 200 μl 40% (wt/vol) trichloroacetic acid (TCA) to the reaction mixture. After incubation at room temperature for 15 min, the mixture was centrifuged at 13,000 × *g* for 10 min, and the absorbance of the supernatant at 335 nm (*A*_335_) was measured in a 1-cm light-path cell. One unit (U) of azocaseinolytic activity was defined as the amount of enzyme required to increase the *A*_335_ by 0.01 per minute under the conditions described above.

### Sodium Dodecyl Sulfate-Polyacrylamide Gel Electrophoresis (SDS-PAGE) and Immunoblot Analysis

Sodium dodecyl sulfate-polyacrylamide gel electrophoresis (SDS-PAGE) was performed using the glycine-Tris (King and Laemmli, 1971) or Tricine-Tris (Schägger et al., 1988) buffer systems. Unless otherwise indicated, protein samples were precipitated with 20% (wt/vol) TCA, washed with ice-cold acetone, and then subjected to electrophoresis on 12.5% gels without prior heat treatment to prevent self-degradation of the protease during sample preparation or electrophoresis. Recombinant ΔNP and PDZ were produced in *E. coli* and served as antigens for the generation of antibodies in rabbits. Antisera against ΔNP (anti-ΔNP) and PDZ (anti-PDZ) were prepared and used for immunoblot analysis as described previously (Cheng et al., 2009). In some cases, the His-tag monoclonal antibody (Novagen) was used for immunoblot analysis as described previously (Cheng et al., 2009).

### Cross-Linking with Glutaraldehyde

Cross-linking of proteins was carried out according to the method of Wang and Guo (2003) with slight modification. Briefly, cross-linking was performed at approximately 0.1 mg/ml protein and 0.001% glutaraldehyde in 50 mM potassium-phosphate buffer (pH 7.5), and incubated for 4 h at room temperature. The reaction was terminated with 0.15 volume 0.5 M glycine. Cross-linked proteins were boiled in loading buffer and then subjected to SDS-PAGE analysis.

### N-terminal Sequencing

After separation by SDS-PAGE, proteins were electroblotted onto a polyvinylidene difluoride membrane and stained with Coomassie Brilliant Blue R-250. The target protein bands were excised and subjected to N-terminal amino acid sequence analysis using a PPSQ-33A protein sequencer (Shimadzu, Kyoto, Japan).

## Results

### Characterization of Recombinant HtrAw Produced in *E. coli*.

**FIGURE 1:**
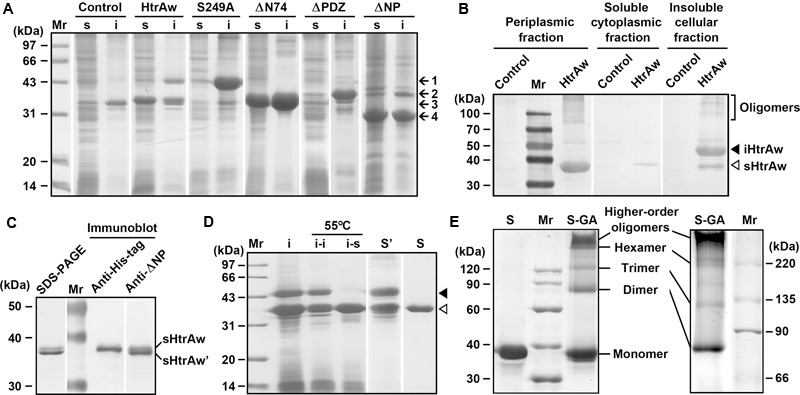
**Production and purification of recombinant proteins expressed in *Escherichia coli*. (A)** SDS-PAGE analysis of the soluble (s) and insoluble (i) cellular fractions of *E. coli* harboring a blank vector (Control) or an expression plasmid for HtrAw or its variants. Arrows indicate the positions of iHtrAw and S249A (1), ΔPDZ (2), sHtrAw and ΔN74 (3), and ΔNP (4) on the gel. **(B)** Localization of recombinant proteins. The periplasmic, soluble cytoplasmic, and insoluble cellular fractions of *E. coli* harboring a blank vector (Control) or an expression plasmid for HtrAw were subjected to anti-His-tag immunoblot analysis. **(C)** SDS-PAGE and immunoblot analyses of the sample of HtrAw short form purified from soluble cellular fraction of *E. coli* by His-tag affinity chromatography. The positions of sHtrAw and its C-terminal truncated form (sHtrAw′) on the gel are indicated. **(D)** Purification of sHtrAw. The insoluble cellular fraction (i) of *E. coli* producing HtrAw was suspended in buffer A and incubated at 55°C for 2 h. The soluble (i–s) and insoluble (i–i) fractions of the suspension were separated by centrifugation, and sHtrAw in the soluble fraction was purified by His-tag affinity chromatography in the presence of 8 M urea (S). Denatured iHtrAw and sHtrAw were co-purified from the insoluble cellular fraction by His-tag affinity chromatography in the presence of 8 M urea (S′). Closed and open arrowheads indicate the positions of iHtrAw and sHtrAw, respectively, on the gel. **(E)** Sodium Dodecyl Sulfate-Polyacrylamide Gel Electrophoresis (SDS-PAGE) analysis of cross-linked proteins. Purified and refolded sHtrAw (S) and glutaraldehyde cross-linked sHtrAw (S-GA) were subjected to SDS-PAGE analysis on a 10% (left panel) or 7.5% (right panel) gel.

HtrAw shares 28% sequence identity with *E. coli* DegS, 36% identity with *B. subtilis* HtrA, 40% identity with *B. subtilis* HtrB, 25% identity with *M. tuberculosis* HtrA2, and 22% identity with human HtrA2 (Supplementary Figure S1). Recombinant HtrAw (44 kDa) with a fused C-terminal His-tag was produced in *E. coli*. The 44-kDa intact HtrAw (named iHtrAw) and a 36-kDa protein were found predominantly in the insoluble and soluble cellular fractions, respectively (**Figure 1A**). The 36-kDa protein was detected with the anti-His-tag antibody, and was primarily located in the periplasmic fraction (**Figure 1B**), indicating that it was a short form (named sHtrAw) of iHtrAw. In contrast, an active-site variant (S249A) of HtrAw was primarily localized in the insoluble cellular fraction as the intact form (**Figure 1A**), suggesting that the active site is involved in the conversion of iHtrAw to sHtrAw. The first four residues of purified sHtrAw (see below) were determined to be SVEV by N-terminal sequencing, indicating that the N-terminal domain is autoprocessed at the peptide bond Ile^74^-Ser^75^ (Supplementary Figure S1).

**FIGURE 2:**
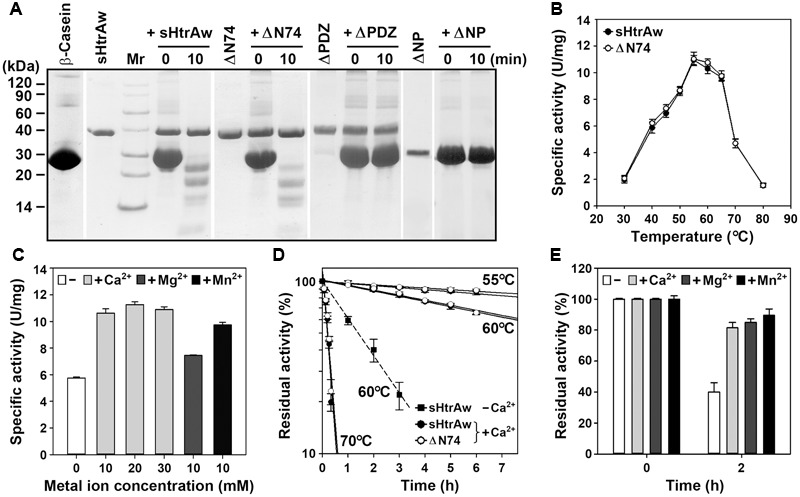
**Properties of HtrAw and its variants. (A)** Hydrolysis of β-casein by enzymes. The reaction was carried out at 55°C in buffer A (50 mM Tris-HCl, 10 mM CaCl_2_, pH 7.5) containing purified enzyme (20 μg/ml) and β-casein (200 μg/ml) for the time periods indicated, and then the samples were subjected to Tricine-SDS-PAGE analysis. **(B)** Temperature dependence of enzyme activity. The azocaseinolytic activities of the enzymes were determined in buffer A at the indicated temperatures. **(C)** Effects of metal ions on enzyme activity. The azocaseinolytic activity of sHtrAw was determined at 55°C in buffer B (50 mM Tris-HCl, pH 7.5) in the absence (–) or presence (+) of 10 to 30 mM CaCl_2_, 10 mM MgCl_2_, or 10 mM MnCl_2_. **(D,E)** Heat-inactivation profiles. The enzymes (75 μg/ml) were incubated at different temperatures in buffer A (+Ca^2+^) or buffer B (–Ca^2+^) for the time intervals indicated **(D)**. Purified sHtrAw (75 μg/ml) was also incubated in buffer B at 60°C for 2 h in the absence (–) or presence (+) of 10 mM CaCl_2_, MgCl_2_, or MnCl_2_ **(E)**. The azocaseinolytic activity was determined at 55°C. Residual activity is expressed as a percentage of the original activity of each enzyme sample. The values are expressed as means ± SD (bars) of three independent experiments.

The short form of HtrAw in the soluble cellular fraction of *E. coli* was purified by His-tag affinity chromatography, but SDS-PAGE analysis of the purified sample revealed two bands with similar, but not identical, molecular weights (**Figure 1C**). The larger one (sHtrAw) was detected by both anti-ΔNP and anti-His-tag antibodies, whereas the smaller one (sHtrAw′) was detected only by anti-ΔNP antibodies (**Figure 1C**). This indicates that the fused His-tag was processed from sHtrAw′. The evidence of co-elution of sHtrAw′ with sHtrAw by His-tag affinity chromatography suggests that the two proteins can form a non-covalent complex. The iHtrAw and sHtrAw proteins were simultaneously isolated from the insoluble cellular fraction of *E. coli* by His-tag affinity chromatography in the presence of 8 M urea (**Figure 1D**, lane S′). We found that sHtrAw proteins could be partially solubilized from the insoluble cellular fraction of *E. coli* after incubation at 55°C for 2 h in buffer A (**Figure 1D**, lane i–s). Solubilized sHtrAw was purified by His-tag affinity chromatography in the presence of 8 M urea to produce a single band on an SDS-PAGE gel (**Figure 1D**, lane S), and the purified sample was refolded and used for enzyme characterization. A glutaraldehyde cross-linking experiment demonstrated that sHtrAw could form dimer, trimer, hexamer, and higher order oligomers (**Figure 1E**). Purified sHtrAw was able to hydrolyze β-casein (**Figure 2A**), and exhibited an optimum temperature of 55°C using azocasein as the substrate (**Figure 2B**). Ca^2+^, Mg^2+^, and Mn^2+^ enhanced the activity of sHtrAw (**Figure 2C**), but Cu^2+^ and Zn^2+^ inactivated the enzyme (data not shown). In the presence of 10 mM CaCl_2_, sHtrAw was a rather thermostable protein, as it retained approximately 90% or 70% of the original activity after incubation at 55°C or 60°C, respectively, for 6 h (**Figure 2D**). Conversely, in the absence of CaCl_2_, this enzyme retained only approximately 20% of the original activity following incubation at 60°C for 3 h (**Figure 2D**). Additionally, Mg^2+^ and Mn^2+^ improved thermostability of sHtrAw (**Figure 2E**).

In order to investigate the roles of the N-terminal domain and the PDZ domain in enzyme function, the N-terminal domain-deletion variant ΔN74, PDZ-deletion variant ΔPDZ, and the variant ΔNP that lacks these two domains were constructed and produced in *E. coli* (**Figure 1A**). Purified ΔN74 hydrolyzed β-casein (**Figure 2A**) with a temperature optimum and heat inactivation profile similar to sHtrAw (**Figures 2B,D**), suggesting that the N-terminal domain is not required for enzyme folding, activity, or thermostability. In contrast to sHtrAw and ΔN74, ΔPDZ and ΔNP were unable to hydrolyze β-casein (**Figure 2A**), indicating that the PDZ domain is critical for enzymatic activity of HtrAw.

### Substrate-Induced Autoprocessing of the N-terminal Domain

**FIGURE 3:**
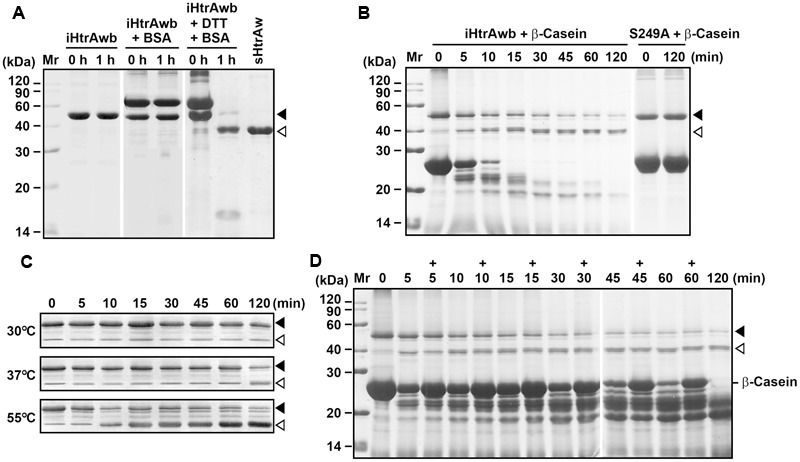
**Substrate-induced autoprocessing of the N-terminal domain. (A,B)** Effects of substrates on enzyme autoprocessing. Purified iHtrAwb or S249A (20 μg/ml) was incubated alone or with (+) BSA (50 μg/ml) or β-casein (200 μg/ml) in the absence or presence (+) of 10 mM DTT in buffer A at 55°C for the time periods indicated, and then the samples were subjected to SDS-PAGE analysis. The samples incubated with 10 mM DTT were loaded with a two-fold increase in protein concentration compared to samples without DTT. **(C)** The effect of temperature on enzyme autoprocessing. Purified iHtrAwb (20 μg/ml) was incubated with β-casein (200 μg/ml) at different temperatures for the time periods indicated, and then the samples were subjected to immunoblot analysis using anti-ΔNP antibodies. **(D)** The effect of full-length substrate on enzyme autoprocessing. The reaction mixture containing iHtrAwb (20 μg/ml) and β-casein (200 μg/ml) was incubated at 55°C for different time periods. At the time points indicated (+), fresh β-casein (100 μg/ml) was added to the reaction mixture. The samples were subjected to SDS-PAGE analysis. Closed and open arrowheads indicate the positions of iHtrAwb and its short form on the gel, respectively.

Our next objective was to probe the mechanism for the conversion of intact HtrAw to its short form. As mentioned above, a portion of recombinant HtrAw proteins with a C-terminal His-tag produced in *E. coli* underwent N-terminal truncation, and iHtrAw was unable to be separated from sHtrAw via His-tag affinity chromatography (**Figure 1D**, lane S′) due to the presence of His-tags at their C-termini. In order to prepare purified intact HtrAw, a recombinant HtrAw (named HtrAwb) fused with an N-terminal His-tag was produced in *E. coli*, and its intact form (iHtrAwb) was purified from the insoluble cellular fraction by His-tag affinity chromatography in the presence of 8 M urea. The refolded iHtrAwb was incubated at 55°C for 1 h, but the protein remained unprocessed during the incubation (**Figure 3A**), indicating that iHtrAwb itself is unable to convert to the short form. In the presence of BSA, iHtrAwb also remained unprocessed, and BSA was not degraded; however, incubation of iHtrAwb with BSA in the presence of dithiothreitol (DTT) resulted in conversion of the intact form to the short form, accompanied by degradation of BSA (**Figure 3A**). Incubation with β-casein also led to the conversion of iHtrAwb to the short form, while the active-site variant S249A remained unprocessed under the same conditions (**Figure 3B**). These results demonstrate that denatured (e.g., reduced BSA) or unstructured (e.g., β-casein) protein substrates can induce autoprocessing of the N-terminal domain of HtrAw. When fresh, intact β-casein was continuously added to the reaction mixture, the conversion of iHtrAwb to the short form was not delayed (**Figure 3D**), indicating that continuous presence of intact substrates does not inhibit autoprocessing of the N-terminal domain of HtrAw. In addition, the β-casein-induced autoprocessing of the N-terminal domain was accelerated with an increase in incubation temperature (**Figure 3C**), reflecting the thermophilic nature of HtrAw.

In order to investigate whether the N-terminal domain of HtrAw is autoprocessed intramolecularly or intermolecularly, denatured S249A (fused with a C-terminal His-tag) and iHtrAwb (fused with an N-terminal His-tag) were mixed and refolded. The co-refolded sample was then incubated with β-casein at 55°C. SDS-PAGE analysis revealed that the amount of short form increased with extension of the incubation time (Supplementary Figure S2). Moreover, the protein bands of short forms were detected by anti-His-tag immunoblot (Supplementary Figure S2). Notably, after removal of the N-terminal domain, the fused His-tag was retained only in the short form of S249A rather than that of iHtrAwb, thus the short form proteins detected by anti-His-tag immunoblot were derived from S249A. As the inactive variant S249A was unable to autoclave its N-terminal domain in the presence of β-casein (**Figure 3B**), clearly the N-terminal domain of S249A in the co-refolded sample was cleaved by iHtrAwb and/or its short form in an intermolecular manner.

In another experiment, separately refolded S249A and iHtrAwb were mixed and incubated with β-casein at 55°C. Although short forms were generated during the incubation, as revealed by SDS-PAGE analysis, only a trace amount of short forms could be detected by anti-His-tag immunoblot (Supplementary Figure S2). This result suggests that most short form proteins generated in the mixture of separately refolded proteins were derived from iHtrAwb. Furthermore, refolded S249A could not be processed into its short form by active sHtrAw in the presence of β-casein (Supplementary Figure S2). Based on the finding that HtrAw is able to form oligomers, we interpret these results to indicate that the cleavage sites in oligomers formed by S249A proteins themselves are inaccessible to the active site of iHtrAwb or sHtrAw.

HtrAs have a conserved substrate-binding site in their PDZ domains (Supplementary Figure S1) (Krojer et al., 2002, 2010; Zeth, 2004). In order to investigate the role of the PDZ domain in autoprocessing of the N-terminal domain, the four residues (Tyr^302^ to Ile^305^) (Supplementary Figure S1) of the substrate-binding site in the PDZ domain of iHtrAwb were simultaneously mutated to Ala, generating a variant Mut4S. When incubated at 55°C, MutS4 proteins could be converted to lower molecular weight products either in the presence or absence of β-casein (Supplementary Figure S3). However, only a small amount of N-terminal domain-truncated short form was detected by anti-PDZ immunoblot, and a low-level β-casein degradation was observed (Supplementary Figure S3). Conversely, most of the lower molecular weight products were detected by anti-ΔNP antibodies, but not by anti-PDZ antibodies (Supplementary Figure S3), suggesting that the PDZ domains of most Mut4S proteins were cleaved. This is in contrast to iHtrAwb, wherein substrates induce autoprocessing of the N-terminal domain to yield an active short form composed of the catalytic domain and the PDZ domain. These results indicate that the substrate-binding site in the PDZ domain plays an important role in substrate-induced autoprocessing of the N-terminal domain of HtrAw.

**FIGURE 4:**
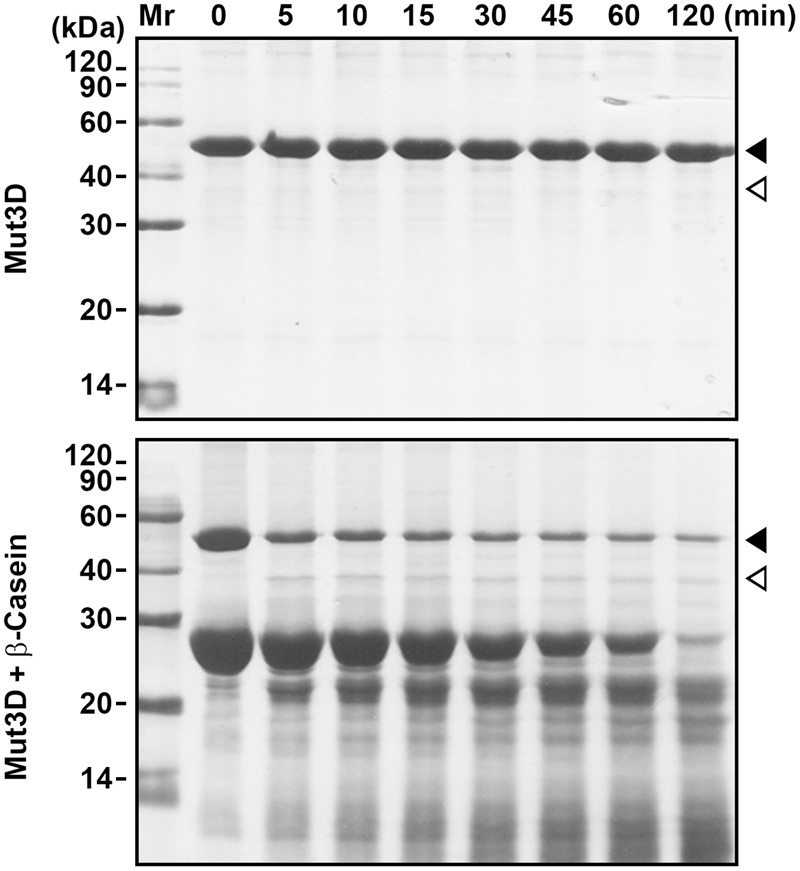
**Mutation of Asp^224^, Asp^226^, and Asp^228^ in the Dx[DN]xDG motif affects autoprocessing of HtrAw.** Purified Mut3D (40 μg/ml) was incubated alone or with β-casein (200 μg/ml) in buffer A at 55°C for the time periods indicated, and then the samples were subjected to SDS-PAGE. Closed and open arrowheads indicate the positions of the intact and short forms on the gel, respectively.

HtrAw contains a Dx[DN]xDG motif (Asp^224^-Gly^229^), which is not present in other known HtrAs (Supplementary Figure S1). In a variety of unrelated proteins, the Dx[DN]xDG motif is known to bind calcium (Rigden et al., 2011). The three Asp residues (Asp^224^, Asp^226^, and Asp^228^) (Supplementary Figure S1) in the Dx[DN]xDG motif of iHtrAwb were simultaneously mutated to Ala, generating a variant Mut3D. Although Mut3D could be converted to the short form at 55°C in the presence of β-casein, but the conversion efficiency and β-casein degradation level in the Mut3D sample (**Figure 4**) were lower than that in the iHtrAwb sample (**Figure 3B**), suggesting that the Ca^2+^-binding Dx[DN]xDG motif is important for structural stability and/or enzyme activity. This is consistant with the finding that Ca^2+^ improved thermostability and activity of sHtrAw (**Figures 2C–E**).

### The Effect of HtrAw on Heat Resistance of an *htrA*/*htrB* Double Deletion Mutant of *B. subtilis* DB104.

**FIGURE 5:**
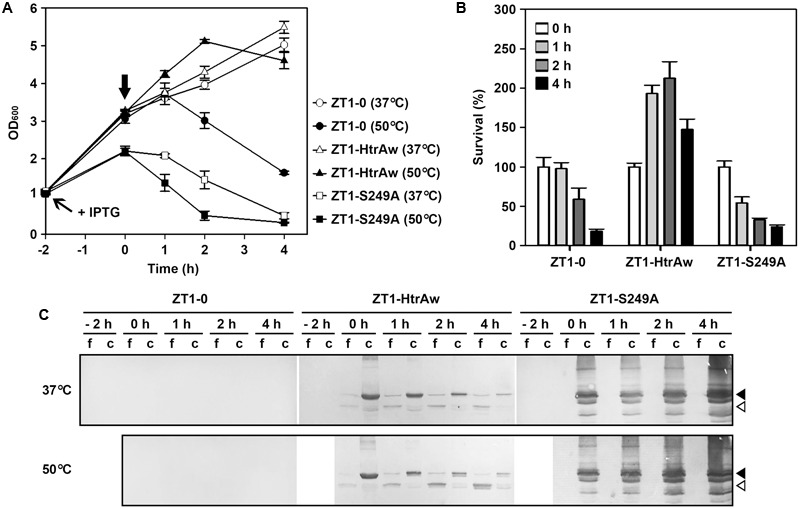
**The effect of HtrAw on heat resistance of *Bacillus subtilis*. (A,B)** Growth and survival of *B. subtilis* strain ZT1 harboring a blank vector (ZT1-0) or an expression vector for HtrAw (ZT1-HtrAw) or S249A (ZT1-S249A). The cultures (OD_600_ = ∼1.0) of the strains grown in LB medium at 37°C were supplemented with 0.4 mM IPTG to induce protein expression for 2 h at 37°C. The cultures were then continually grown at 37°C or shifted to 50°C at time zero (indicated by a vertical arrow in **A**) for increasing periods of time. Culture growth was monitored by measuring OD_600_ **(A)**. Survival was determined by plating appropriate dilutions of samples taken at the time points indicated. The number of viable cells of each strain present at time zero was set at 100% **(B)**. Values are expressed as means ± SD (bars) of three independent experiments. **(C)** Immunoblot analysis of recombinant proteins produced in *B. subtilis* strains. The culture supernatants (f) and cell pellets (c) collected at the time points indicated were subjected to anti-His-tag immunoblot analysis. The amount of sample loaded into each lane was calculated based on the growth curve to ensure that the supernatant proteins or cell pellets were from the same number of cells. Closed and open arrowheads indicate the positions of the intact and short forms on the gel, respectively.

HtrA and HtrB contribute to the heat resistance of *B. subtilis*, and disruption of both *htrA* and *htrB* makes the bacterium more sensitive to thermal stress (Noone et al., 2001). In order to investigate whether HtrAw performs a function similar to *B. subtilis* HtrA and HtrB, an *htrA/htrB* double deletion mutant of *B. subtilis* DB104 (strain ZT1) was constructed and used as the host to produce HtrAw and its active-site variant S249A. The strain ZT1 harboring a blank vector (strain ZT1-0) or an expression vector for HtrAw (strain ZT1-HtrAw) or S249A (ZT1-S249A) was grown in LB medium at 37°C. After induction with IPTG for 2 h, the cultures were continually grown at 37°C or shifted to 50°C for increasing time periods. A comparison of the growth profiles of these strains are shown in **Figure 5A**. The strain ZT1-HtrAw showed a growth profile similar to that of ZT1-0 at 37°C, but it grew better than the latter after being shifted to 50°C, suggesting that having an HtrAw-encoding gene facilitates the ability of the bacterium to grow at an elevated temperature. In contrast, ZT1-S249A grew much poorer than ZT1-0 at either 37°C or 50°C. ZT1-S249A growth was slower than that of ZT1-0 or ZT1-HtrAw after IPTG induction at 37°C, indicating that production of S249A has a negative effect on growth of the bacterium.

In order to compare the heat resistance of strains ZT1-0, ZT1-HtrAw, and ZT1-S249A, we determined their survival after being shifted to 50°C (**Figure 5B**). The results showed that ZT1-HtrAw was much more resistant to heat stress than ZT1-0 and ZT1-S249A, indicating that functional HtrAw improves heat tolerance of the bacterium. It is also evident than the strain ZT1-S249A was more sensitive to heat stress than ZT1-0, reflecting a negative effect of S249A on thermotolerance of the bacterium.

Immunoblot analysis revealed that, after IPTG induction for 2 h at 37°C, iHtrAw was localized predominantly in the cell pellet fraction of the ZT1-HtrAw culture, and minor sHtrAw proteins were present in the culture supernatant (**Figure 5C**, lane 0 h). During an extended period of cultivation at either 37°C or 50°C, the amount of sHtrAw in the culture supernatant increased (**Figure 5C**, lanes 1–4 h). In contrast, under the same cultivation conditions, S249A accumulated only in the cell pellet fraction of ZT1-S249A and suffered degradation, likely due to the bacterial membrane protease(s) (**Figure 5C**). These results suggest that recombinant HtrAw can be released from the cytoplasmic membrane of *B. subtilis* via autoprocessing of the TMS-containing N-terminal domain. Moreover, the ratio of free sHtrAw to cell-associated iHtrAw proteins was higher at 50°C than at 37°C (**Figure 5C**). This implies that cleavage of the N-terminal domain of cell-associated iHtrAw is more pronounced at elevated temperatures, in agreement with *in vitro* data, wherein the autoprocessing of the TMS-containing N-terminal domain was temperature-dependent (**Figure 3C**).

### Production and Localization of HtrAw in *Brevibacillus* sp. WF146

**FIGURE 6:**
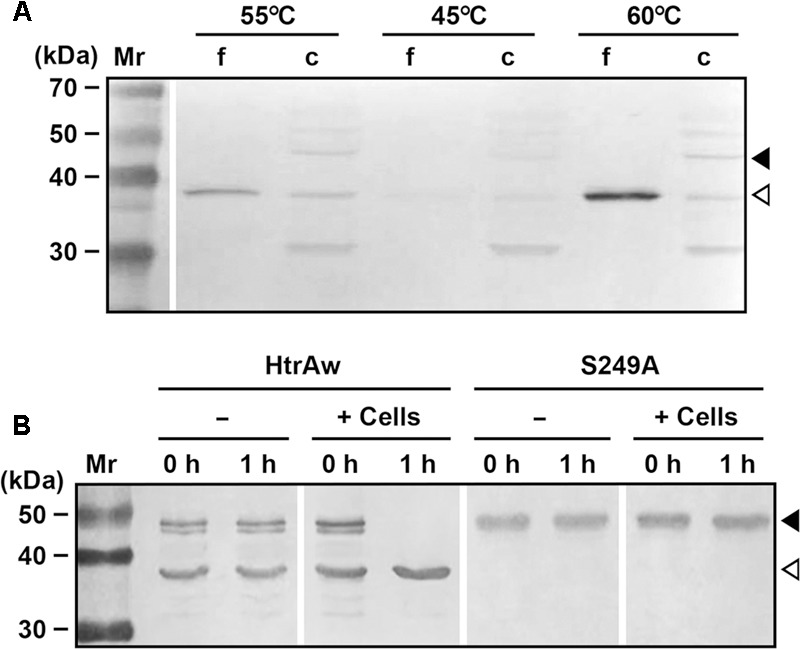
**Production and processing of HtrAw in *Brevibacillus* sp. WF146. (A)** The effect of temperature on HtrAw production. The strain WF146 was grown in LB medium at the temperatures indicated. The supernatants (f) and cell pellets (c) of exponentially growing cultures were subjected to immunoblot analysis using anti-ΔNP antibodies. The amount of sample loaded in each lane was calculated based on the growth curve to ensure that the supernatant proteins or cell pellets were from the same number of cells. **(B)** The effect of strain WF146 cells on HtrAw processing. The exponentially growing WF146 strain cells were thoroughly washed with buffer A. The refolded samples (10 μg/ml) of HtrAw (contains both iHtrAw and sHtrAw) and S249A were incubated either alone (–) or with (+) strain WF146 cells (2.0 × 10^8^ cells/ml) at 55°C in buffer A. At the time points indicated, supernatants of the samples were collected and subjected to anti-His-tag immunoblot analysis. Closed and open arrowheads indicate the positions of the intact and short forms on the gel, respectively.

*Brevibacillus* sp. WF146 grows optimally at approximately 58°C in LB medium (Wu et al., 2004). In order to investigate the localization and production profile of HtrAw in the strain WF146, exponentially growing cultures of the bacterium at 45, 55, and 60°C were subjected to immunoblot analysis. Intact HtrAw was detected only in the cell pellet fraction grown at all temperatures tested (**Figure 6A**), implying that the intact form is able to anchor to the cytoplasmic membrane via the TMS. The cell-associated HtrAw could be released from the membrane as a short form by removing the TMS-containing N-terminal domain, as evidenced by the presence of free sHtrAw in the culture supernatants (**Figure 6A**). Meanwhile, the short form was also detected in the cell pellet fractions (**Figure 6A**), likely associated with the cell via an as yet unknown mechanism. The other bands detected in the cell pellet may have resulted from degradation of the HtrAw monomer and oligomer (**Figure 6A**). Notably, the amount of HtrAw, particularly that of the free short form, increased remarkably as the growth temperature was increased from 45 to 60°C (**Figure 6A**), suggesting that production of HtrAw in the strain WF146 is temperature dependent. Based on the finding of the substrate-induced autoprocessing of the N-terminal domain of recombinant HtrAw, we hypothesized that cellular factor(s) (e.g., cell surface proteins) of the strain WF146 may be involved in the conversion of intact HtrAw to its short form. To test this possibility, the sample containing both iHtrAw and sHtrAw, which was prepared from the insoluble cellular fraction of *E. coli* via His-tag affinity chromatography in the presence of 8 M urea (**Figure 1D**, lane S′), was refolded and then incubated either alone or with *Brevibacillus* sp. WF146 cells at 55°C. After a 1-h incubation, iHtrAw was converted into sHtrAw in the presence of the cells, but remained unchanged in the absence of the cells (**Figure 6B**). In contrast, the active-site variant S249A was unable to convert to the short form in the presence or absence of the cells (**Figure 6B**). These results suggest that cellular factors of the strain WF146 can induce autoprocessing of the N-terminal domain of HtrAw.

## Discussion

In this study, an HtrA-like protease (HtrAw) from thermophilic *Brevibacillus* sp. WF146 was characterized. Similar to other HtrAs from Gram-positive bacteria, intact HtrAw is composed of a TMS-containing N-terminal domain, a trypsin-like protease catalytic domain, and a C-terminal PDZ domain. HtrAw also shares common characteristics with other HtrAs, such as the N-terminal domain-mediated membrane anchoring of the enzyme, the capacity to form oligomers, and the requirement of the PDZ domain for enzymatic activity. In addition, HtrAw can increase the heat resistance of an *htrA*/*htrB* double deletion mutant of *B. subtilis* DB104, and its production level in *Brevibacillus* sp. WF146 increased as the growth temperature increased. These data suggest that HtrAw performs a function similar to other known HtrAs such as *B. subtilis* HtrA and HtrB (Noone et al., 2001; Darmon et al., 2002).

Metal ions have different effects on HtrAs from different species. The activity of *Borrelia burgdorferi* HtrA is inhibited by Zn^2+^, Mn^2+^, or Cu^2+^, and the activity of *E. coli* DegP or human HtrA1 was also affected by Zn^2+^ (Russell et al., 2015). In contrast, Ca^2+^ and Mg^2+^ increase the activity of HtrA from *Synechocystis* sp. PCC 6803 (Huesgen et al., 2011). Here, we found that Ca^2+^, Mg^2+^, and Mn^2+^ enhanced the activity and thermostability of HtrAw, while Cu^2+^ and Zn^2+^ inactivated the enzyme. Interestingly, HtrAw contains a Ca^2+^-binding Dx[DN]xDG motif that contributes to structural stability and/or enzyme activity of the enzyme. Notably, the Dx[DN]xDG motif is absent in known HtrAs from mesophiles (Supplementary Figure S1). In this context, the Dx[DN]xDG motif-mediated Ca^2+^-binding is an important mechanism for adaptation of HtrAw to high temperatures.

Although the N-terminal domain of HtrAw acts as a membrane anchor, iHtrAw can be converted into sHtrAw by autocleavage of the peptide bond Ile^74^-Ser^75^ downstream of the TMS (Supplementary Figure S1). The conversion of an intact form to a short form has been reported for other HtrAs with a TMS-containing N-terminal domain. The cleavage sites of N-terminal domains of *B. subtilis* HtrA (Antelmann et al., 2003), *M. tuberculosis* HtrA2 (Mohamedmohaideen et al., 2008), and human HtrA2 (Suzuki et al., 2001) are also located several residues downstream of the TMS (Supplementary Figure S1). *B. subtilis* HtrA (Antelmann et al., 2003) and HtrB (Zweers et al., 2009) can be released into the culture medium as an N-terminal domain truncated short form. In *B. subtilis*, membrane proteases PrsW and RasP are known for intramembrane cleavage of TMSs and shedding ectodomain of membrane proteins, respectively (Dalbey et al., 2012). However, the disruption of *rasP* or *prsW* did not prevent the release of short forms of HtrA and HtrB into the culture medium (Zweers et al., 2009). Additionally, disruption of the genes encoding eight extracellular proteases (AprE, Bpr, Epr, Mpr, NprB, NprE, Vpr, and WprA) also does not block the release of HtrA and HtrB into the culture medium, although the two HtrAs are prone to proteolysis by WprA, NprB, and AprE (Krishnappa et al., 2014). These results raise the possibility that the TMS-containing N-terminal domains in *B. subtilis* HtrA and HtrB are cleaved autocatalytically. N-terminal domain truncated HtrA2 of *M. tuberculosis*, which is a Gram-positive species, but shares some features with Gram-negative bacteria including the presence of a periplasmic space, has been detected in the culture medium, likely resulting from autocatalytic cleavage of the extracytoplasmic portion of the enzyme (Skeiky et al., 1999; Mohamedmohaideen et al., 2008; White et al., 2010). An investigation of cellular distribution of *M. tuberculosis* HtrA2 revealed that intact wild-type HtrA2 localized to the cytoplasmic membrane and the cell wall, and that its active-site variant localized predominately to the cell wall. Thus, it is proposed that HtrA2 traffics from the cytoplasm through the cytoplasmic membrane to the cell wall, where it is autoprocessed and eventually shed into the culture medium as a truncated form (White et al., 2011). It is unclear, however, how membrane-anchored intact HtrA2 is released and transported from the cytoplasmic membrane to the cell wall of *M. tuberculosis*. In the case of human HtrA2, the TMS-containing N-terminal domain of the wild-type protein rather than that of its active-site variant can be cleaved off, suggesting that conversion of full-length human HtrA2 to its N-terminal truncated form is an autocatalytic process (Gray et al., 2000; Suzuki et al., 2001). Therefore, autoprocessing of the TMS-containing N-terminal domain seems to be a common feature shared by HtrAs despite their different origins.

Importantly, we found that HtrAw itself was unable to convert to the short form, but required denatured or unstructured protein substrates for autoprocessing of the N-terminal domain in an intermolecular manner. There are at least two possible explanations for the requirement of substrates for autoprocessing the N-terminal domain of HtrAw. One possibility is that intact HtrAw itself is inactive and requires substrates to form an active conformation, thereby allowing the N-terminal domain to be autoprocessed. However, we are uncertain whether intact HtrAw is active or not because it could be converted into an active short form immediately after mixing with substrates such as reduced BSA and β-casein (**Figure 3**). Future experimental endeavors (e.g., construction of an intact HtrAw variant resistant to autocleavage) are required to address this issue. It is also possible that intact HtrAw has a properly arranged catalytic triad, but its autocleavage site (e.g., Ile^74^-Ser^75^) was inaccessible to the active site in the absence of substrates. The presence of protein substrates may lead to a structural adjustment of intact HtrAw, making the autocleavage site readily accessible to the active site for hydrolysis. In either case, substrates appear to trigger a structural change that facilitates autocleavage of the N-terminal domain of HtrAw.

The mature form of *E. coli* DegP undergoes partial degradation as a consequence of autocleavage at two sites within a long LA loop of the catalytic domain, and hydrolysis of protein substrates is required for the autocleavage event, producing two N-terminal truncated short forms (Skórko-Glonek et al., 1995, 2003; Kim et al., 1999). In resting DegP hexamer, the LA loop of one subunit protrudes into the active site of another subunit, and the substrate hydrolysis product allosterically stimulates enzymatic activity via binding to the PDZ domain (Jomaa et al., 2009). The autocleavage of HtrAw differs from that of DegP in several aspects. First, HtrAw has a much shorter LA loop compared to DegP, and the autocleavage site of HtrAw is located between the N-terminal domain and the catalytic domain rather than within the LA loop (Supplementary Figure S1). Secondly, the only apparent requirement for accumulation of the N-terminal truncated short form of DegP *in vitro* is the hydrolysis of protein substrates, as the continuous presence of full-length substrate delays the autocleavage process, and the short form accumulates only late in the degradation reaction (Jomaa et al., 2009). However, the autocleavage of HtrAw was not delayed when full-length substrate was added continuously. Thirdly, the autocleavage of DegP affects enzymatic activity and the cleaved region is required for proper folding of the enzyme (Jomaa et al., 2009), whereas the N-terminal domain of HtrAw was not necessary for enzyme folding, activity, and thermostability. The data regarding DegP suggest that the autocleavage process is to eliminate excess DegP from the cell once stress conditions are overcome (Jomaa et al., 2009). In contrast, autoprocessing of the N-terminal domain of HtrAw yielded a functional short form. Despite these differences, HtrAw and DegP appear to be similar regarding the requirement of structural adjustment for autocleavage to occur. Substrate-triggered allosteric activation is a common feature of HtrAs, and protein substrates or their hydrolysis products can act as allosteric activators by binding to the PDZ domain (Krojer et al., 2008a, 2010). Consistent with these reports, we found that the PDZ domain of HtrAw was indispensable for enzymatic activity and autoprocessing of the N-terminal domain. Moreover, mutations in the substrate binding site of the PDZ domain affected the conversion of intact HtrAw to its short form. These data suggest that PDZ-mediated substrate binding promotes the structural adjustment of HtrAw, which allows for autocleavage.

In *Brevibacillus* sp. WF146, HtrAw exists in both a cell-associated intact form and a cell-free short form, and the amount of short form increased as the growth temperature increased. Based on the *in vitro* data of substrate-induced autoprocessing of the TMS-containing N-terminal domain, we propose that HtrAw is initially anchored to the cytoplasmic membrane by its TMS, and the interaction between the intact form and protein substrates in the culture medium leads to the release of the short form into the medium by autoprocessing of the N-terminal domain. Meanwhile, cellular factors of the strain WF146 (e.g., surface-exposed and secreted proteins) can also induce autoprocessing of the N-terminal domain of HtrAw. The TMS-mediated membrane anchoring of HtrAw may have the advantage of increasing the local enzyme concentration around the cells. Once protein substrates reach the cell surface and interact with HtrAw to trigger the autocleavage process, the resulting short form is released from the membrane to freely access and degrade the substrates surrounding the cells.

## Author Contributions

FZ, XY, YanW, and YasW conducted the experiments; and FZ, X-FT, and BT analyzed and interpreted the results and contributed to writing the paper.

## Conflict of Interest Statement

The authors declare that the research was conducted in the absence of any commercial or financial relationships that could be construed as a potential conflict of interest.
