# Supplementary material for: Release of an HtrA-Like Protease from the Cell Surface of Thermophilic Brevibacillus sp. WF146 via Substrate-Induced Autoprocessing of the N-terminal Membrane Anchor
Source: Front Microbiol. 2017 Mar 21;8:481. doi: 10.3389/fmicb.2017.00481 (PMC5359297; doi:10.3389/fmicb.2017.00481)
Supplement: Supplementary file 1 [file Table_1.PDF]

TABLE S1 Oligonucleotide primers used in this study

Primer	Oligonucleotide sequence (5′ to 3′)[a]
ΔA-5F	GTTAAGAAGCTTATACGTTTGGATAATCTGACAT
ΔA-5R	ACGGCCTGAGGCATTATGTCCATGTTCACTCCGTTTCT
ΔA-3F	AGAAACGGAGTGAACATGGACATAATGCCTCAGGCCGT
ΔA-3R	GTCAAGGAATTCATGATTAGAGCCGTACCAGTTATG
ΔB-5F	GTTAAGAAGCTTACTAGATAAATGGTGTATGACAAG
ΔB-5R	GTTCAGCTTTTTGTTTTTCGTTCTTACACTCCTTTAAC
ΔB-3F	GTTAAAGGAGTGTAAGAACGAAAAACAAAAAGCTGAAC
ΔB-3R	GTCAAGGAATTCTGGCGATTGGCGGACAGCCTGTTG
Aw-F	GGAATTCCAT**ATG**GGTTTTTACGATGATATG
Aw-R	GTTAAGAAGCTTCCGCTGCGGCGGATTGGTCA
Awb-F	GGAATTCCAT**ATG***CACCACCACCACCACCAC*GGTTTTTACGATG
Awb-R	GTTAAGAAGCTT**TTA**CCGCTGCGGCGGATTGGTCA
ΔN-F	GGAATTCCAT**ATG**TCCGTCGAAGTCAATAC
ΔP-R	GTTAAGAAGCTTTTTCAACTGGCCGTACTG
PDZ-F	GGAATTCCAT**ATG**CGACCGTATCTGGGCATTATTC
NotI-F	AGGAGGAAGGATCA**ATG**GCGGCCGCATTCAAAAACGAAAG
NotI-R	TCGTTTTTGAATGCGGCCGCCATTGATCCTTCCTCCTTTAAT
Aw-BF	AAGGAAAAAAGCGGCCGCTGGTTTTTACGATGATATGGCGCAT
Aw-BR	GCTAGTCTAGA**TTA***GTGGTGGTGGTGGTGGTG*CCGCTGCGGCGGATT
SA-F	GAACGCTGGCGGCGCGCTGGTCAACATCT
SA-R	GCCAGCGTTCCCCGGGTTGAT
YA-F	AGTTGAAACGACCGGCTGCAGCAGCTATTCCTTACG
YA-R	TCAAATCGTAAGGAATAGCTGCTGCAGCCGGTCGT
DA-F	CTCCATGCCGATTGCCGTGGCTCAAGCCGGGCAGGAT
DA-R	GCTCCCAGTCATCCTGCCCGGCTTGAGCCACGGCAATC

[a]Underlined sequences indicate the restriction enzyme sites. Italicized sections indicate the His-tag-encoding sequences. Translation initiation and termination codons are shown in bold. Open boxes indicate the mutated nucleotides.
