# Supplementary material for: Release of an HtrA-Like Protease from the Cell Surface of Thermophilic Brevibacillus sp. WF146 via Substrate-Induced Autoprocessing of the N-terminal Membrane Anchor
Source: Front Microbiol. 2017 Mar 21;8:481. doi: 10.3389/fmicb.2017.00481 (PMC5359297; doi:10.3389/fmicb.2017.00481)
Supplement: Supplementary file 2 [file Table_2.PDF]

TABLE S2 Primer pairs, templates, and restriction sites used in plasmid construction

Plasmid	Primer pair	Template	Restriction site
pNNB194-*ΔhtrA*	ΔA-5F/ΔA-5R	Genomic DNA (DB104)	-
	ΔA-3F/ΔA-3R	Genomic DNA (DB104)	-
	ΔA-5F/ΔA-3R	Joined DNA fragment[a]	*Hin*d III-*Eco*R I
pNNB194-*ΔhtrB*	ΔB-5F/ΔB-5R	Genomic DNA (DB104)	-
	ΔB-3F/ΔB-3R	Genomic DNA (DB104)	-
	ΔB-5F/ΔB-3R	Joined DNA fragment[a]	*Hin*d III-*Eco*R I
pET26b-*iHtrAw*	Aw-F/Aw-R	Genomic DNA (WF146)	*Nde* I-*Hin*d III
pET26b-*iHtrAwb*	Awb-F/Awb-R	Genomic DNA (WF146)	*Nde* I-*Hin*d III
pET26b-*ΔN74*	ΔN-F/Aw-R	pET26b-*iHtrAw*	*Nde* I-*Hin*d III
pET26b-*ΔPDZ*	Aw-F/ΔP-R	pET26b-*iHtrAw*	*Nde* I-*Hin*d III
pET26b-*ΔNP*	ΔN-F/ΔP-R	pET26b-*iHtrAw*	*Nde* I-*Hin*d III
pET26b-*PDZ*	PDZ-F/Aw-R	pET26b-*iHtrAw*	*Nde* I-*Hin*d III
pET26b-*S249A*	SA-F/SA-R	pET26b-*iHtrAw*	-
pET26b-*YLGI/A*	YA-F/YA-R	pET26b-*iHtrAwb*	-
pET26b-*DDD/A*	DA-F/DA-R	pET26b-*iHtrAwb*	-
pHT43a	NotI-F/NotI-R	pHT43	-
pHT43a-*iHtrAw*	Aw-BF/Aw-BR	Genomic DNA (WF146)	*Not* I-*Xba* I
pHT43a-*S249A*	SA-F/SA-R	pHT43a-*iHtrAw*	-

[a]The joined DNA fragment was obtained by overlap extension PCR of the two PCR products of approximately 500-bp DNA sequences upstream of the 5′ end and downstream of the 3′ end of the target gene.
