# Supplementary material for: Release of an HtrA-Like Protease from the Cell Surface of Thermophilic Brevibacillus sp. WF146 via Substrate-Induced Autoprocessing of the N-terminal Membrane Anchor
Source: Front Microbiol. 2017 Mar 21;8:481. doi: 10.3389/fmicb.2017.00481 (PMC5359297; doi:10.3389/fmicb.2017.00481)
Supplement: Supplementary file 4 [file Image_2.PDF]

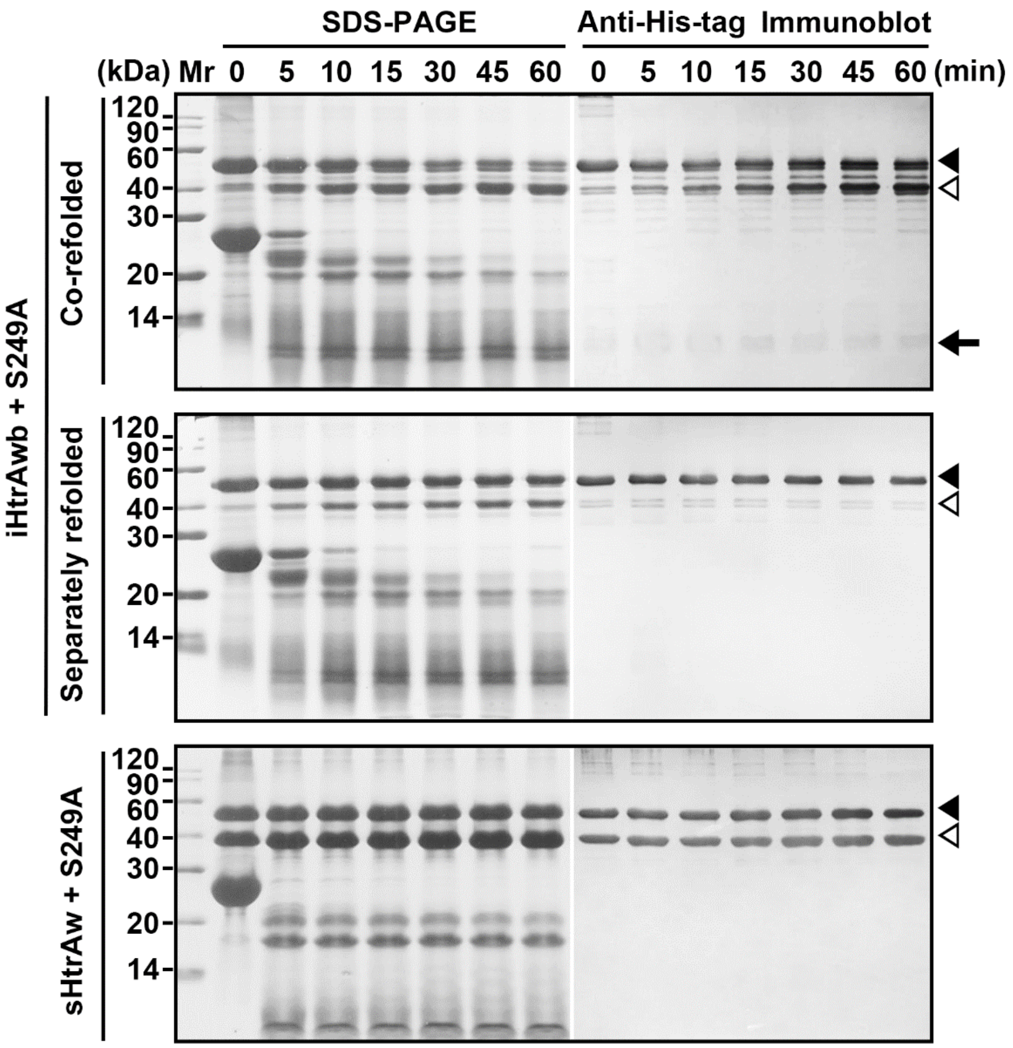

FIGURE S2 Intermolecular processing of HtrAw. The 8 M urea-denatured iHtrAwb (20 μg/ml) and S249A (20 μg/ml) were co-refolded or refolded separately by dialysis against buffer A at 4°C. The co-refolded and separately refolded samples were incubated with β-casein (200 μg/ml) at 55°C. Refolded S249A (40 μg/ml) was mixed with sHtrAw (40 μg/ml) and incubated with β-casein (200 μg/ml) at 55°C in buffer A. At the time points indicated, aliquot samples were taken and subjected to SDS-PAGE and anti-His-tag immunoblot analyses. Closed and open arrowheads indicate the positions of the intact form and the short form on the gel, respectively. The cleaved N-terminal domain is indicated by an arrow.
