# Supplementary material for: Release of an HtrA-Like Protease from the Cell Surface of Thermophilic Brevibacillus sp. WF146 via Substrate-Induced Autoprocessing of the N-terminal Membrane Anchor
Source: Front Microbiol. 2017 Mar 21;8:481. doi: 10.3389/fmicb.2017.00481 (PMC5359297; doi:10.3389/fmicb.2017.00481)
Supplement: Supplementary file 5 [file Image_3.PDF]

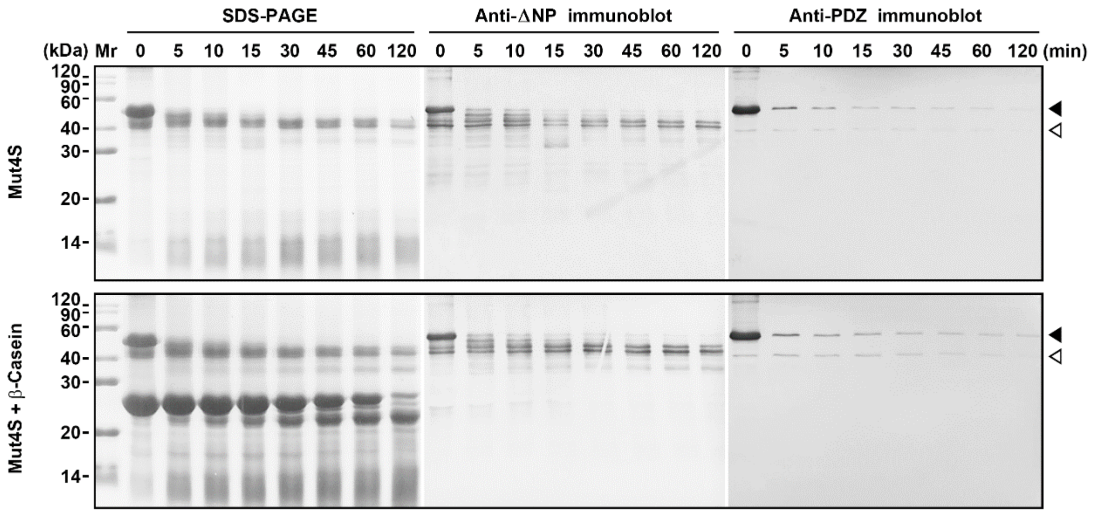

FIGURE S3 The role of a substrate-binding site in the PDZ domain on autoprocessing of HtrAw. Purified Mut4S (40 μg/ml) was incubated alone (upper panel) or with β-casein (200 μg/ml) (lower panel) in buffer A at 55°C for the time periods indicated, and then the samples were subjected to SDS-PAGE and immunoblot analyses using anti-ΔNP or anti-PDZ antibodies. Closed and open arrowheads indicate the positions of the intact and short forms on the gel, respectively.
