# Supplementary material for: Release of an HtrA-Like Protease from the Cell Surface of Thermophilic Brevibacillus sp. WF146 via Substrate-Induced Autoprocessing of the N-terminal Membrane Anchor
Source: Front Microbiol. 2017 Mar 21;8:481. doi: 10.3389/fmicb.2017.00481 (PMC5359297; doi:10.3389/fmicb.2017.00481)
Supplement: Supplementary file 3 [file Image_1.PDF]

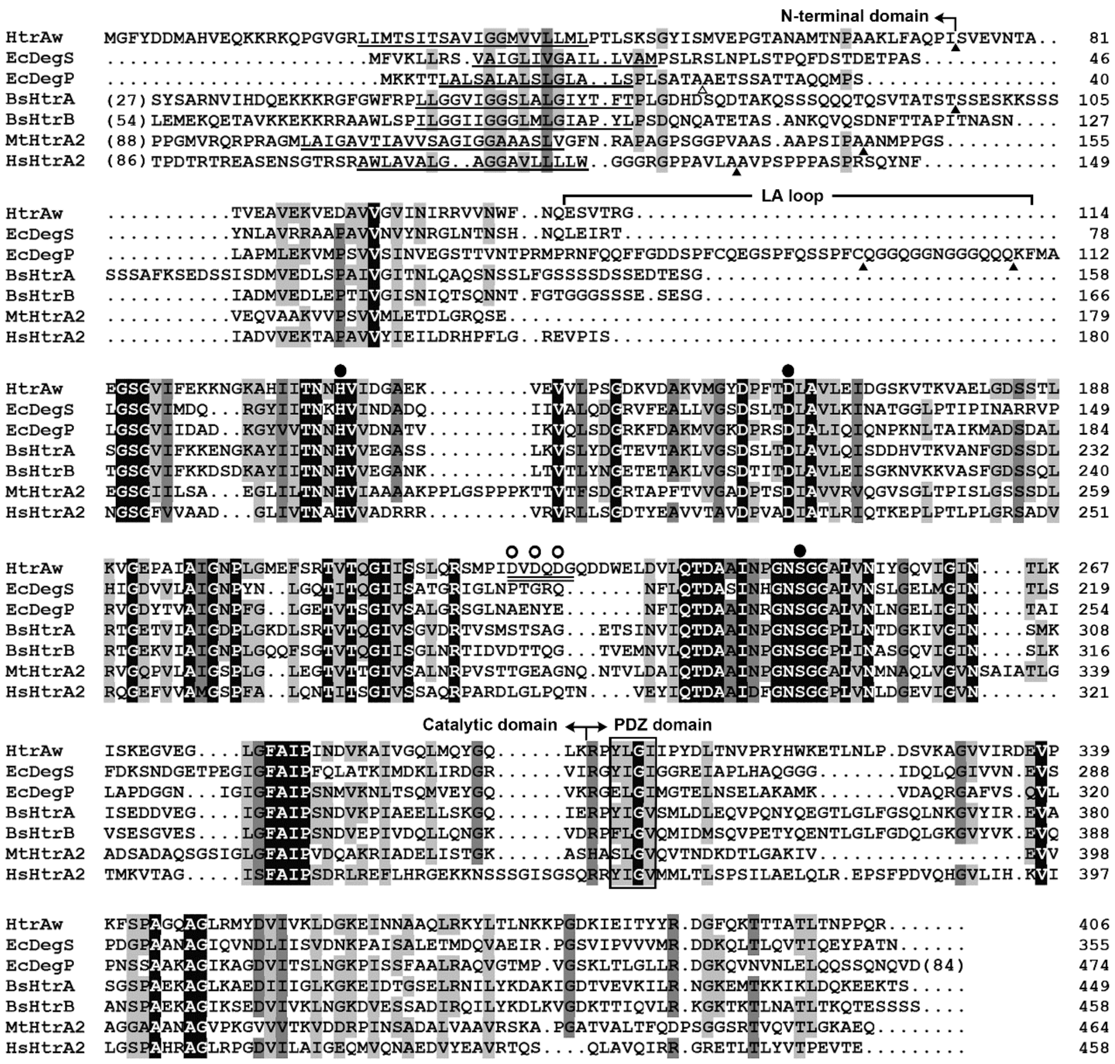

FIGURE S1 Alignment of amino acid sequences of HtrAw (WP_029100535) and *E. coli* DegS (EcDegS) (AAC44006) and DegP (EcDegP) (BAL37467), *B. subtilis* HtrA (BsHtrA) (O34358) and HtrB (BsHtrB) (Q9R9I1), *M. tuberculosis* HtrA2 (MtHtrA2) (CCP43733), and human HtrA2 (HsHtrA2) (O43464). The N-terminal domain, the catalytic domain, the PDZ domain, and the LA loop are indicated. Filled circles mark the catalytic triad residues. Transmembrane sequences are underlined. Open and closed arrowheads indicate the cleavage site of the signal peptide of *E. coli* DegP and the autocleavage sites identified in some HtrAs, respectively. Boxed residues indicate the substrate-binding site in the PDZ domain. The Ca^{2+}-binding Dx[DN]xDG motif is double-underlined, and open circles indicate residues that were mutated.
